# Extracellular domain of CD98hc is required for early murine development

**DOI:** 10.1186/2045-3701-1-7

**Published:** 2011-02-25

**Authors:** Yukiyasu Sato, Rachel A Heimeier, Cuiling Li, Chuxia Deng, Yun-Bo Shi

**Affiliations:** 1 Section on Molecular Morphogenesis, Laboratory of Gene Regulation and Development, Program in Cellular Regulation and Metabolism (PCRM), NICHD, NIH, Bethesda, Maryland, 20892, USA; 2 Department of Gynecology and Obstetrics, Kyoto University Graduate School of Medicine, Sakyo-ku, Kyoto 606-8507, Japan; 3 Institute of Environmental Medicine (IMM), Karolinska Institutet (KI), Nobels väg 13, S-171 77, Stockholm, Sweden; 4 Mammalian Genetics Section, GDDB, NIDDK, NIH, Bethesda, Maryland, 20892, USA

## Abstract

**Background:**

The multifunctional protein CD98 heavy chain (CD98hc, Slc3a2) associates with integrin β1 through its cytoplasmic and transmembrane domains and the CD98hc-mediated integrin signaling is required for maintenance of ES cell proliferation. CD98hc-null mice exhibit early post-implantation lethality similar to integrin β1-null mice, supporting the importance of its interaction with integrin β1. On the other hand, the extracellular domain of CD98hc interacts with L-type amino acid transporters (LATs) and is essential for appropriate cell surface distribution of LATs. LATs mediate the transport of amino acids and other molecules such as thyroid hormone. In this respect, CD98hc may also affect development via these transporters.

**Results:**

In this study, mice were generated from embryonic stem (ES) cell line (PST080) harboring a mutant CD98hc allele (CD98hc^Δ/+^). Expression of the CD98hc mutant allele results in ΔCD98hc-β geo fusion protein where extracellular C-terminal 102 amino acids of CD98hc are replaced with β geo. Analyses of PST080 ES cells as well as reconstituted frog oocytes demonstrated that ΔCD98hc-β geo fusion protein preserved its ability to interact with integrin β1 although this mutant protein was hardly localized on the cell surface. These findings suggest that ΔCD98hc-β geo protein can mediate integrin signaling but cannot support amino acid transport through LATs. CD98hc^Δ/+ ^mice were normal. Although some of the implantation sites lacked embryonic component at E9.5, all the implantation sites contained embryonic component at E7.5. Thus, CD98hc^Δ/Δ ^embryos are likely to die between E7.5 and E9.5.

**Conclusions:**

Considering that CD98hc complete knockout (CD98hc^-/-^) embryos are reported to die shortly after implantation, our findings suggest potential stage-specific roles of CD98hc in murine embryonic development. CD98hc may be essential for early post-implantation development by regulating integrin-dependent signaling, while the other function of CD98hc as a component of amino acid transporters may be required for embryonic development at later stages.

## Background

CD98 heavy chain (CD98hc, Slc3a2) is a multifunctional membrane protein composed of an N-terminal cytoplasmic domain, intermediate transmembrane domain, and C-terminal extracellular domain. CD98hc was originally identified as an activated antigen of lymphocytes in human and mouse [1]. Subsequent studies revealed at least two distinct functions of CD98hc. First, CD98hc associates with one of several L-type amino acid transporters (LATs) to form heterodimeric amino acid transporter (HAT) complexes that are also capable of transporting other molecules such as thyroid hormone [2-6]. LATs have multiple membrane-spanning domains and are believed to provide the transport activity of HAT complexes, whereas CD98hc regulates the functional cell surface localization of LATs [7,8]. The extracellular domain of CD98hc is necessary for its interaction with LATs to support amino acid transport [9]. Indeed, Broer et al. showed that only 68-amino-acid deletion from the C-terminus of human CD98hc is sufficient to disrupt proper translocation of LATs to the plasma membrane, resulting in severe impairment of the transporter activity [7]. Secondly, CD98hc associates with integrin β1 and modulates the function of integrin β1 to promote cell adhesion and migration [10]. The cytoplasmic and transmembrane domains are required and sufficient for this association [9].

Embryonic stem (ES) cell lines that lack CD98hc (CD98hc^-/-^) gene have an impaired ability to spread on fibronectin or laminin and are susceptible to cell death [11]. Furthermore, CD98hc^-/- ^ES cells rarely form teratocarcinoma in nude mice due to severely impaired proliferative activity. These lethal phenotypes of CD98hc^-/- ^ES cells are completely rescued by concomitant overexpression of chimeric CD98hc protein whose extracellular domain is replaced by that of unrelated transmembrane protein [11], indicating that cytoplasmic and transmembrane domains of CD98hc that mediate integrin β1 interaction are sufficient to support ES cell proliferation. Thus, lack of CD98hc-mediated integrin signaling is a likely cause of reduced proliferation in CD98hc-null ES cells.

CD98hc^-/- ^embryos have been reported to die shortly after implantation [12]. Although the precise cause has not yet been determined, impaired proliferative activity of CD98hc-null ES cells due to defective integrin signaling may contribute to this early lethality. Similarly, integrin β1-null embryos exhibited retarded growth of inner cell mass and die shortly after implantation [13,14]. In this respect, the cytoplasmic and transmembrane domains of CD98hc that mediate integrin β1 interaction and modulation may play a critical role in early post-implantation development.

Here, we examined the role of the extracellular domain of CD98hc, which is dispensable for ES cell proliferation [11] but is essential for amino acid transport via LATs [7,9], in murine development. For this purpose, we generated mutant mice with ES cell line PST080, harboring a mutant CD98hc allele that encodes a truncated form of CD98hc protein (ΔCD98hc) fused to β galactosidase and neomycin phosphotransferase II (β geo). In this ΔCD98hc-β geo fusion protein, the extracellular 102 amino acids from the C terminal end of CD98hc are replaced by β geo. We show that this mutant protein preserves its ability to interact with integrin β1. On the other hand, ΔCD98hc-β geo protein mostly localizes in the cytoplasm and thus may be unable to support amino acid transport. *In vivo *analysis suggests that the homozygous mutant (CD98hc^Δ/Δ^) embryos die between E7.5 and E9.5. These findings underscore the possible importance of amino acid transport system in early murine development.

## Results

### CD98hc mRNA is expressed in multiple adult and fetal tissues

**Figure 1:**
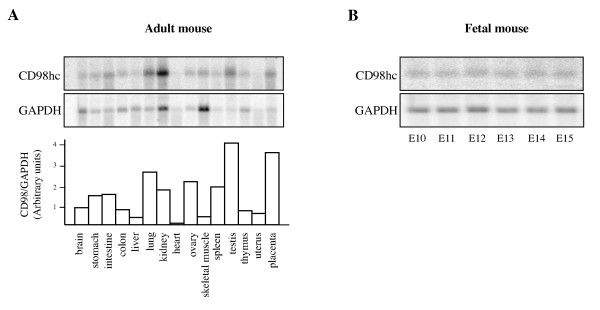
**CD98hc mRNA expression in adult and fetal mice**. A mouse mixed tissue Northern blot (A) and a mouse embryo Northern blot (B) were hybridized with a CD98hc cDNA probe and a GAPDH probe as a loading control. Bar graph shows the relative CD98hc mRNA expression levels normalized to GAPDH mRNA levels.

Northern blot analysis revealed that CD98hc is expressed in a wide variety of adult tissues (Figure 1A). High levels of expression are observed in lung, kidney, spleen, ovary, testis and placenta. On the other hand, expression is weak in liver, heart, and skeletal muscle. CD98hc mRNA is constitutively expressed in mouse embryos from E10 through E15 (Figure 1B). These results suggest that CD98hc play roles in various organs and during embryogenesis.

### Generation of CD98hc gene trap mice

**Figure 2:**
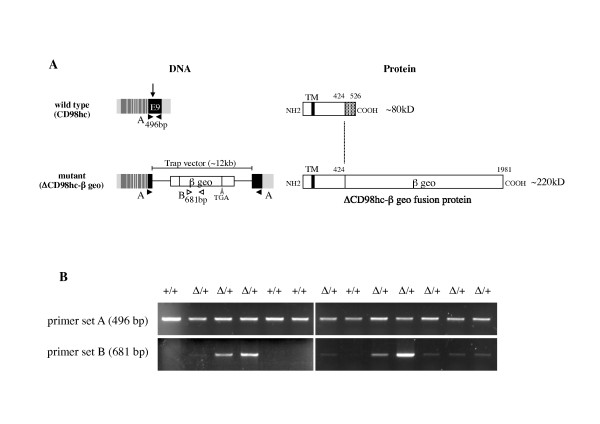
**Insertional mutation in CD98hc**. **A**. Schematic representation of the mutation in CD98hc. Nine exons are shown as boxes with untranslated regions at 5' and 3' ends. The gene trap vector was inserted within the translated region of exon 9 (arrow) to yield an in-frame fusion transcript with β-geo. Binding sites for oligonucleotide primer sets A (black arrowheads) and B (white arrowheads) used for PCR genotyping are indicated. CD98hc is a ~80 kD membrane protein, which is composed of N-terminal cytoplasmic, transmembrane (TM), and C-terminal extracellular domains. The mutant allele is expected to produce a ~220 kD protein whose C-terminal 102 amino acids are replaced with β-geo. **B**. Representative results of genotyping PCR of 3-week-old offspring from a heterozygous (CD98hc^Δ/+^) intercross. Primer set A, which amplifies whole translated region of exon 9 (496 bp), yields the expected PCR product from wild type CD98hc allele (+). Primer set B, which amplifies a part of β-geo sequence (681 bp), yields PCR products only from the mutant allele (Δ). Note that primer set A yields the 496-bp band that shows the presence of wild type CD98hc allele in all the offspring. Note, the amount of genomic DNA used for each PCR was not quantified, and therefore quantitative levels cannot be directly compared.

Mice were generated from ES cells containing an insertional mutation (gene trap) in exon 9 of the mouse CD98hc gene. As shown in Figure 2A, the insertion of gene trap vector generates an in-frame fusion transcript between a reporter gene (β geo) and partially deleted CD98hc gene. In the resultant protein, 102 amino acids from the C-terminus were deleted and replaced by β geo. Two sets of specific primers were designed to distinguish the wild type and mutant allele (Figure 2A). Primer set A spans the entire coding region of exon 9 (496 bp), and primer set B amplifies 681 bp within β galactosidase sequence. Because the size of the trap vector is extremely large (~12 kb), primer set A cannot yield any specific PCR product from the mutant allele. On the other hand, primer set B cannot bind to wild type allele. Therefore, wild type animals (CD98hc^+/+^) should produce only the 496 bp band from set A, homozygous mutant animals (CD98hc^Δ/Δ^) should produce only the 681 bp band from set B, whereas heterozygous animals (CD98hc^Δ/+^) should produce both bands from sets A and B (representative example in Figure 2B).

### ΔCD98hc-β geo fusion protein is translated in heterozygous (CD98hc^Δ/+^) mice

**Figure 3:**
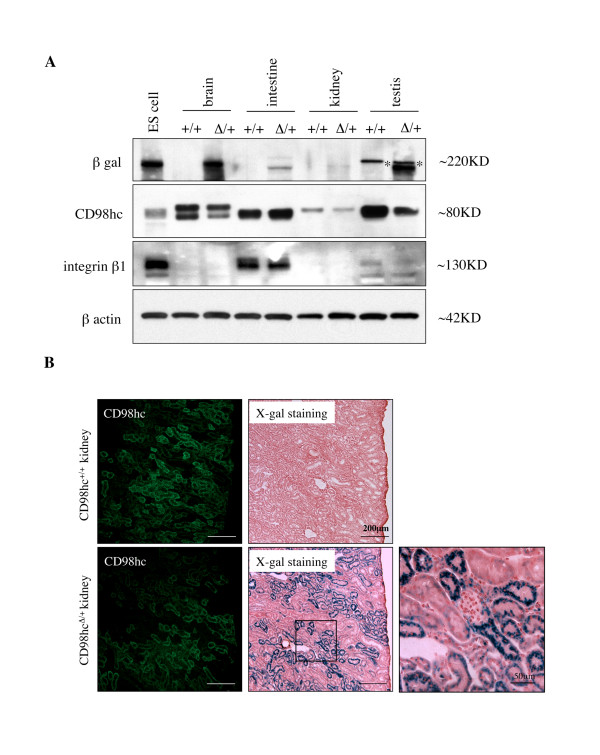
***In vivo *expression of ΔCD98hc-β geo fusion protein**. **A**. ΔCD98hc-β geo fusion protein is expressed in CD98hc^Δ/+ ^mouse. Protein was extracted from CD98hc^Δ/+ ^ES cells (PST080) or various tissue samples harvested from wild type (CD98hc^+/+^) and heterozygous mutant (CD98hc^Δ/+^) mice for Western blotting. Anti-β-galactosidase (β gal) antibody yields ~220 kD bands specific to CD98hc^Δ/+ ^samples, while anti-CD98hc antibody yields ~80 kD bands in all samples. Note that the different mobility of the proteins from different tissues is likely due to different posttranslational modifications. The expression of integrin β1, which can interact with CB98hc, was detected in ES cells, intestine, and testis samples, but not in brain or kidney sample. The β actin was analyzed as a loading control. *, nonspecific bands. **B**. The expression pattern of ΔCD98hc-β geo fusion protein mimics that of CD98hc protein in CD98hc^Δ/+ ^mouse. Kidney sections from CD98hc^+/+ ^and CD98hc^Δ/+ ^mice were immunostained with anti-CD98hc antibody or stained with X-gal to localize β-galactosidase activity that represents ΔCD98hc-β geo fusion protein. Note that distribution of immunoreactive CD98hc is the same as that of β-galactosidase activity in CD98hc^Δ/+ ^kidneys. Lower right panel shows the magnified image of boxed area indicated in lower middle panel.

To verify the expression of ΔCD98hc-β geo fusion protein in heterozygotic (CD98hc^Δ/+^) mice, protein was extracted from brain, intestine, kidney and testis for Western blotting. As shown in Figure 3A, CD98hc expression was detectable in all tissues examined. Anti-β galactosidase polyclonal antibody yielded specific band of the expected size for ΔCD98hc-β geo fusion protein (~220 kD) in CD98hc^Δ/+ ^tissues but not in CD98hc^+/+ ^counterparts. Furthermore, the relative expression levels of ΔCD98hc-β geo fusion protein in different organs were similar to those of the wild type CD98hc protein. Expression of integrin β1, which is known to interact with CD98hc, was detectable in intestine and testis, but not in brain or kidney (Figure 3A). Consistently, RT-PCR analysis of the transcripts also showed coexpression of the mRNAs of ΔCD98hc-β geo and CD98hc in different organs of heterozygous mice (Additional file 1).

### The expression pattern of ΔCD98hc-β geo fusion protein mimics that of CD98hc protein in CD98hc^Δ/+ ^animals

To further analyze the expression pattern of ΔCD98hc-β geo fusion protein, we chose the kidney for immunohistochemical comparison of the expression of ΔCD98hc-β geo fusion protein and wild type CD98hc *in vivo *since the renal tubules makes it easy to compare their spatial expression patterns in an organ. Thus, kidney sections were stained with X-gal for detection of β-galactosidase activity and neighboring section was immunostained with anti-CD98hc monoclonal antibody (clone H202-141). As described in more detail below, this antibody had a very weak affinity for the CD98hc-β geo fusion protein and thus mainly detected CD98hc (Table 1). As shown in Figure 3B, immunoreactive CD98hc was detected in proximal renal tubules in CD98hc^Δ/+ ^as well as CD98hc^+/+ ^kidney. β galactocidase activity, which reflects the presence of ΔCD98hc-β geo fusion protein, was detected in the CD98hc^Δ/+ ^but not in the CD98hc^+/+ ^kidney. More importantly, the distribution of β galactosidase activity was the same as that of immunoreactive CD98hc in CD98hc^Δ/+ ^kidney (Figure 3B). Since the transcription of ΔCD98hc-β geo were driven by the same promoter as wild type CD98hc, it was not surprising that the expression profile of ΔCD98hc-β geo fusion protein was the same as that of wild type CD98hc. The X-gal staining also revealed that the fusion protein was expressed in different adult tissues, especially in the brain region (Additional file 1), in agreement with the mRNA expression of CD98hc in wild type mouse (Figure 1).

**Table 1:** Immunocytochemical detection of wild type CD98hc, ΔCD98hc,and ΔCD98hc-β geo fusion protein over expressed in frog oocytes.

	anti-CD98hc mAb	anti-CD98hc pAbs		anti-β galactosidase mAb
	(H202-141)	(N-20)	(M-20)	(5A3)
CD98hc	+	+	+	-
ΔCD98hc	+	+	-	-
ΔCD98hc-β geo	+/-	+/-	-	+

N-20 was raised against N-terminal (cytoplasmic) 20 amino acids. M-20 was raised against C-terminal (extracellular) 20 amino acids. mAb, monoclonal antibody; pAb, polyclonal antibody

### ΔCD98-βgeo fusion protein is mainly localized in the cytoplasm

**Figure 4:**
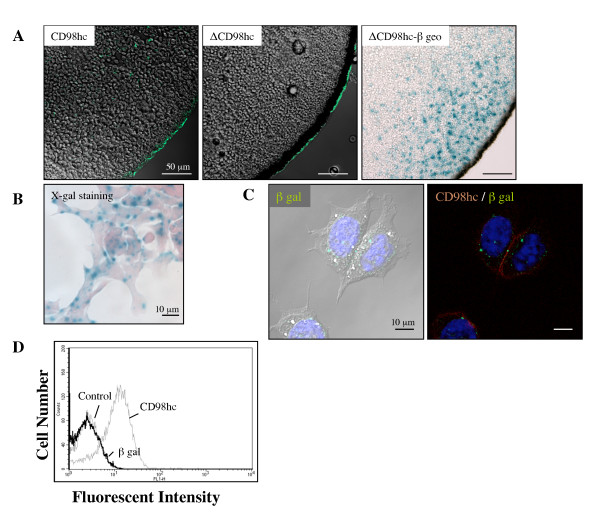
**ΔCD98hc-β geo fusion protein is present mainly in the cytoplasm**. **A**. Wild type CD98hc (CD98hc), mutant CD98 (ΔCD98hc-β geo) or mutant CD98hc without β geo fusion (ΔCD98hc) were expressed in frog oocytes. The oocytes were sectioned and immunostained with anti-CD98hc antibody (green) for detection of CD98hc and ΔCD98hc or stained with X-gal for detection of β-galactosidase (β gal, blue) activity in ΔCD98hc-β geo. ΔCD98hc-β geo was localized mainly in the cytoplasm, although ΔCD98hc was clearly detected on the cell surface. Note that expression of ΔCD98hc-β geo is intense near the cell surface. **B**. X-gal staining of CD98hc^Δ/+ ^ES cells (PST080). β gal activity (blue) that represents ΔCD98hc-β geo protein is mainly localized in the cytoplasm. **C**. PST080 ES cells were double-immunostained with anti-CD98hc (red) and anti-β gal (green) antibodies. Note that ΔCD98hc-β geo expression as detected by anti-β gal antibody was localized mainly in the cytoplasm. Blue staining shows nucleus. **D**. Histogram showing the expression levels of CD98hc and β gal in PST080 ES cells. The ES cells were fixed and immunolabeled with anti-CD98hc or anti-β gal antibody for detection by flow cytometry. No significant cell surface labeling was produced by anti-β gal antibody compared to the control antibody.

To determine the subcellular localization of ΔCD98-βgeo fusion protein, we first used a reconstituted frog oocyte system. The mRNAs of wild type CD98hc, mutant CD98 (ΔCD98hc-β geo) or mutant CD98hc without β geo fusion (ΔCD98hc) were microinjected into the cytoplasm of frog oocytes to express the corresponding proteins. The oocytes were subsequently sectioned and immunostained with anti-CD98hc monoclonal antibody (clone H202-141), anti-CD98hc polyclonal antibodies (N-20 and M-20), or anti-β galactosidase monoclonal antibody (clone 5A3). As summarized in Table 1, wild type CD98hc was recognized by all of three anti-CD98hc antibodies examined. M-20, which was raised against the C-terminal 20 amino acids, could not recognize ΔCD98hc, which lacks the epitope. Interestingly, none of the three anti-CD98hc antibodies could consistently recognize ΔCD98hc-β geo, probably because the large β geo fusion peptide masked the epitopes. ΔCD98hc-β geo protein, however, is clearly detected by anti-β galactosidase monoclonal antibody (Table 1). Expression of CD98hc as well as CD98hc was clearly localized on the cell surface (Figure 4A, left and middle panels). In contrast, ΔCD98hc-β geo fusion protein was mostly localized in the cytoplasm close to the plasma membrane as judged by X-gal staining, with only a low level on the surface (Figure 4A, right panel). Cytoplasmic expression of ΔCD98hc-β geo fusion protein was also confirmed by immunostaining with β galactosidase monoclonal antibody (data not shown).

The subcellular localization of ΔCD98hc-β geo fusion protein in PST080 ES cells (CD98hc^Δ/+^) was also examined either with X-gal staining or with double immunostaining using β galactosidase monoclonal antibody and CD98hc monoclonal antibody. As shown in Figure 4B and 4C, ΔCD98hc-β geo fusion protein was barely detected on the cell surface, whereas CD98hc was detected mainly on the cell surface. Flow cytometry analysis showed that most of the cells had higher levels of surface CD98hc (Figure 4D). As none of the antibodies against CD98hc could consistently bind to ΔCD98hc-β geo fusion protein, we used the anti-β galactosidase monoclonal antibody for the flow cytometry analysis to examine the cell surface expression of ΔCD98hc-β geo fusion protein. No significant cell surface labeling was detected (Figure 4D). These results suggest that most of the fusion proteins were cytoplasmic and only a small fraction reached the cell surface.

### ΔCD98-β geo fusion protein interact with integrin β1

**Figure 5:**
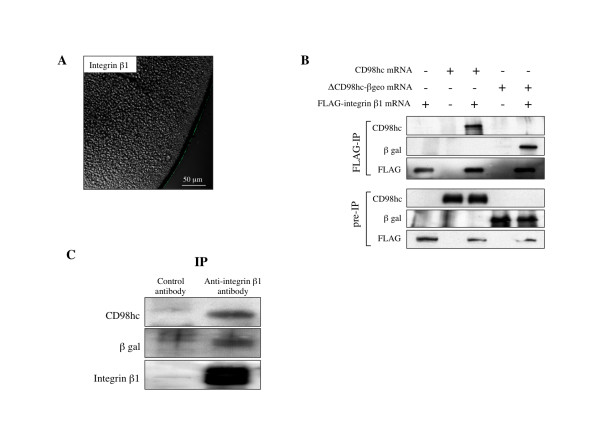
**ΔCD98hc-β geo fusion protein interacts with integrin β1 similarly as wild type CD98hc**. **A**. FLAG-tagged integrin β1 and wild type CD98hc or ΔCD98hc-β geo fusion protein were expressed in frog oocytes. Immunostaining of the sectioned oocytes with anti-integrin β1 antibody verified the cell surface expression (green) of FLAG-tagged integrin β1 protein. **B**. Co-immunoprecipitation analysis was performed with frog oocytes that expressed FLAG-tagged integrin β1 and wild type CD98hc or ΔCD98hc-β geo fusion protein. Anti-FLAG antibody immunoprecipitated FLAG-integrin β1 as expected and also co-immunoprecipitated CD98hc and ΔCD98hc-β geo fusion protein with similar efficiencies. **C**. Co-immunoprecipitation analysis was performed with PST080 ES cells. Anti-integrin β1 antibody pulled down ΔCD98hc-β geo fusion protein or CD98hc together with endogenous integrin β1.

CD98hc interacts with integrin β1 through the cytoplasmic and transmembrane domains and this interaction is essential for ES cell proliferation [9]. To examine whether ΔCD98hc-β geo fusion protein maintains the ability to interact with integrin β1, FLAG-tagged integrin β1 and wild type CD98hc or ΔCD98hc-β geo fusion protein was expressed in frog oocytes. Immunostaining of the sectioned oocytes with anti-integrin β1 antibody verified the cell surface expression of FLAG- integrin β1 protein (Figure 5A). Immunoprecipitation of the FLAG- integrin β1 protein with an anti-FLAG antibody also pulled downΔCD98hc-β geo fusion protein (Figure 5B). Importantly, similar amounts of wild type CD98hc andΔCD98hc-β geo fusion protein were co-immunoprecipitated with integrin β1 (Figure 5B, based on the ratios of the signals for CD98hc and ΔCD98hc-β geo fusion protein in the IP panel to those in the pre-IP panel). Similarly, co-immunoprecipitation analysis with integrin β1 antibody using PST080 ES cells also confirmed the interaction of endogenous integrin β1 with ΔCD98hc-β geo fusion protein (Figure 5C). Thus, while most of the ΔCD98hc-β geo fusion protein was localized in the cytoplasm, sufficient amounts were present near or on the cell surface to interact with integrin β1, consistent with the lack of any apparent defects in PST080 ES cells.

### Homozygous mutant (CD98hc^Δ/Δ^) animals are embryonic lethal

**Figure 6:**
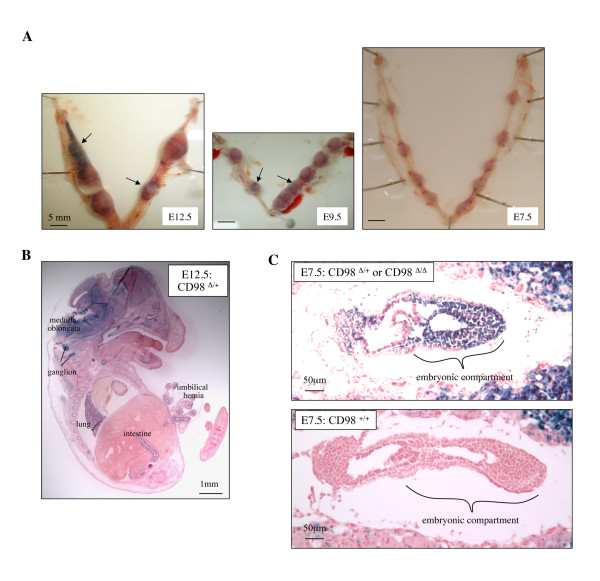
**CD98hc^Δ/Δ^mouse is embryonic lethal between E7.5 and E9.5**. **A**. Pregnant uteri from heterozygous (CD98hc^Δ/+^) intercrosses were dissected at E12.5, E9.5 and E7.5. Some of the implantation sites were apparently smaller at E12.5 and E9.5. These smaller implantation sites were filled with blood clots (indicated by arrows) and appeared to lack embryonic component. In contrast, all the implantation sites were similar in size and contained embryonic component at E7.5, suggesting that CD98hc^Δ/Δ ^is embryonic lethal between E7.5 and E9.5. **B and C**. β-galactosidase activity was visualized by X-gal staining to localize ΔCD98hc-β geo fusion protein in CD98hc^Δ/+ ^embryo at E12.5 (B), either CD98hc^Δ/+ ^or CD98hc^Δ/Δ ^embryo at E7.5 (C, upper panel), and CD98hc^+/+ ^embryo at E7.5 (C, lower panel). At E12.5, the expression Of ΔCD98hc-β geo fusion protein was most prominent in neuronal tissue, and weaker expression was also observed in lung and intestine. At E7.5, prominent expression of ΔCD98hc-β geo fusion protein was observed in the embryonic compartment of the CD98hc^Δ/+ ^or CD98hc^Δ/Δ ^embryo, but not in the CD98hc^+/+ ^embryo.

Heterozygous (CD98hc^Δ/+^) mice were normal in terms of weight, growth, fertility, and macroscopic appearance. No CD98^Δ/Δ ^mouse was found among the offspring from heterozygous intercrosses at the time of weaning (3 weeks after birth) (Figure 2B and Table 2). To determine the time of death of homozygous mice during embryogenesis, the embryos from heterozygous intercrosses were dissected at E12.5 and E9.5. No CD98^Δ/Δ ^embryo was found at these stages (Table 2). Of the implantation sites evaluated at E9.5 and E12.5, about one-third were smaller in size with visible blood clots (Figure 6A and Table 2). Although these resorption sites lacked embryonic component necessary for genotyping, they are likely to be derived from CD98^Δ/Δ ^embryos that had died at earlier stage.

**Table 2:** Genotype distribution of offspring from heterozygous (CD98hc ^Δ/+^)mating

Number of animals of indicated genotype				
Age or embryonic day	CD98hc^+/+^	CD98hc^Δ/+^	CD98hc^Δ/Δ^	Number of resorption site
3 weeks	4/13(31%)	9/13(69%)	0/13(0%)	Not applicable
E12.5	5/14(36%)	9/14(64%)	0/14(0%)	4
E9.5	2/11(11%0	9/11(82%)	0/11(0%)	5
E7.5	4/20(20%)	16/20 (80%)		0

### CD98^Δ/Δ ^embryo die between E7.5 and E9.5

To further investigate the time of death of the homozygous embryos, CD98hc^Δ/Δ ^we analyzed implantation sites at E7.5. In contrast to E12.5 and E9.5, each implantation site was similar in size and contained embryonic components at E7.5 (Figure 6A). Genotyping of these embryos was initially evaluated by immunostaining with anti-CD98hc (M-20) antibody. This antibody cannot react with ΔCD98hc-β geo mutant protein (Table 1), thus embryos devoid of M-20 staining should be homozygous mutants (CD98hc^Δ/Δ^). However, in our hands, this antibody generated significant non-specific binding such that the precise genotyping could not be established with it. On the other hand, X-gal staining of CD98hc^Δ/+ ^embryo at E12.5 revealed prominent expression of CD98hc-β geo fusion protein in neuronal tissue and weaker expression in lung and intestine (Figure 6B). This suggests that CD98hc function in these tissues might be critical for embryonic survival and that the staining could be used for embryo identification. X-gal staining of embryonic sections at E7.5 from 4 pregnant female mice revealed positive staining for 16 embryos and absence of staining in 4 embryos (Figure 6C and Table 2). The former likely represented embryos that were expressing the fusion protein in a heterozygous or homozygous pattern while the latter indicate a total absence of the fusion protein, i.e. wild type embryos. If the homozygous embryos were lethal at E7.5, the ratio of 1:2 between wild type and heterozygous mice would be expected. The observed ratio of 1:4 (Table 2) suggests that although we were unable to detect differences in the X-gal signal intensity among embryos, both heterozygous and homozygous embryos were present at E7.5. Thus, homozygous mutant embryos appear to die between E7.5 and E9.5.

## Discussion

In this study, we have examined the possible significance of the extracellular domain of CD98hc in murine development. The extracellular domain of CD98hc is dispensable for integrin β1 interaction that is essential for maintenance of ES cell proliferation [9-11]. On the other hand, this domain is required for interaction with LATs and translocation of LATs to the plasma membrane [7,9]. For our purpose, we generated mutant mice by using PST080 ES cells harboring mutant CD98hc allele that is expected to be translated into the mutant CD98hc protein (ΔCD98hc-β geo) whose intracellular and transmembrane domains are intact but extracellular 102 amino acids from C terminal end are replaced with β geo. Our results showed that deletion of the extracellular domain resulted in an embryonic lethal phenotype, suggesting the importance of the transporter function of CD98hc in mouse development.

Heterozygous (CD98hc^Δ/+^) mice appeared normal in terms of weight, growth, fertility, and macroscopic appearance. The ratio of heterozygous to wild type offspring from intercrosses of heterozygous animals suggests that all heterozygous embryos developed to full term normally. On the other hand, all the homozygous mutant animals died *in utero*. At E12.5 and E9.5, some of the implantation sites were apparently smaller than the others and were filled with blood clots. At E7.5, all the implantation sites were comparable in size and contained embryonic components. Embryos at this stage have completed gastrulation and are composed of three germ layers, ectoderm, mesoderm, and endoderm. While we were not able to carry out genotyping on such early embryos, X-gal staining revealed a ratio of 1 to 4 for the wild type embryos to homozygous plus heterozygous mutants, suggesting that homozygous mutant survived up to this stage. Thus, it is likely that CD98hc^Δ/Δ ^embryos die between E7.5 and E9.5.

It has been reported that CD98hc^-/- ^embryos die shortly after the implantation [12]. Our mutant embryos appeared to survive at least to E7.5. We showed that the mutant gene had similar expression profiles as the wild type one in heterozygous animals. More importantly, we showed that when the mutant protein was overexpressed in frog oocytes, it was distributed near the plasma membrane and interacted to a similar degree with integrin β1 as wild type CD98hc, although most of the CD98hc-β geo fusion protein was retained in the cytoplasm of the oocytes. These results suggest that CD98hc-β geo fusion protein presumably can still mediate integrin β1 function. As the exact stage for embryonic lethality was not conclusively determined for the CD98hc complete knockouts [12], it is not certain whether CD98hc^Δ/Δ ^embryos survive into later stages of embryonic development than CD98hc^-/- ^embryos. On the other hand, CD98hc associates with integrin β1 and modulates the function of integrin β1 [10,11]. In addition, integrin β1-null embryo exhibits retarded growth of inner cell mass and dies shortly after implantation [13,14]. Thus, it is likely that the preserved ability of ΔCD98hc-β geo fusion protein to interact with integrin β1 allows the homozygous mutant embryos to survive into later stages of embryonic development than CD98hc^-/- ^embryos. Regardless, our findings support the idea that possible gastrulation failure observed in CD98hc^Δ/Δ ^embryos is not mediated by defective integrin β1 function but rather due to the loss of other functions involving an intact extracellular domain of CD98hc.

The extracellular domain of CD98hc is required for the interaction with LATs [7,9]. CD98hc guides LATs to the cell surface to form HAT complexes that are also capable of transporting other molecules such as thyroid hormone [4,5,8]. In the reconstituted frog oocyte system as well as in PST080 ES cells, ΔCD98hc-β geo fusion protein was localized in the cytoplasm. Therefore, it is likely that this mutant protein cannot direct LATs to the cell surface and that functional HAT complexes are disrupted in CD98hc^Δ/Δ ^embryos. Considering the existing evidence supporting the significance of amino acids in early embryonic development [15], it is likely that the impaired amino acid transport could be responsible for the death of the CD98hc^Δ/Δ ^embryos between E7.5 and E9.5.

## Conclusions

We introduced mutant CD98hc protein into mice. This mutant protein preserved its interaction with integrin β1 that could be sufficient to support ES cell proliferation and early embryogenesis. On the other hand, the mutant protein was defective in plasma membrane localization that is required for CD98hc-LAT transporter activity. Homozygous mutants are embryonic lethal between E7.5 and E9.5. These findings suggest stage-specific roles of CD98hc in murine embryonic development. CD98hc may play an essential role for early post-implantation development by regulating integrin-mediated ES cell proliferation, while the other major function of CD98hc as a component of amino acid transporters may be required for subsequent embryonic development.

## Methods

### Antibodies

Anti-mouse CD98hc rat monoclonal antibody (mAb) (clone H202-141) was purchased from Fitzgerald (Concord, MA), two anti-mouse CD98hc goat polyclonal antibodies (pAbs), N-20 and M-20, which were raised against a peptide mapping within an N-terminal cytoplasmic domain and a C-terminal extracellular domain, respectively, were both obtained from Santa Cruz (Santa Cruz, CA). Anti-β galactosidase mouse mAb (clone 5A3) and rabbit pAb were from MBL (Woburn, MA) and Chemicon (Temecula, CA), respectively. Anti-mouse integrin β1 armenian hamster mAb (clone HMbeta1.1) and rat mAb (clone 9EG7) were purchased from AbD Serotec (Raleigh, NC) and BD Pharmingen (San Jose, CA), respectively. Rabbit anti-integrin β1 pAb was from Chemicon. Anti-β actin mouse mAb (clone mAbcam8224) was purchased from Abcam. Anti-FLAG M2 mouse mAb was from Sigma (Saint Louis, MO). Alexa488-conjugated goat anti-mouse IgG pAb and Alexa488-conjugated donkey anti-rat IgG pAb were from Molecular Probes (Eugene, OR). FITC-conjugated donkey anti-goat IgG pAb and rhodamine-conjugated goat anti-rat IgG pAb were from Santa Cruz.

### Generation of CD98hc gene trap mice

Mouse embryonic stem (ES) cell line (PST080, strain 129/Ola) harboring an insertional mutation in *CD98hc *was created in a gene-trapping program, BayGenomics http://baygenomics.ucsf.edu/. The gene-trapping vector, pGT1lxf, was designed to create an in-frame fusion between the 5' exons of the trapped gene and a reporter, β geo (a fusion of β galactosidase and neomycin phosphotransferase II). CD98hc spans 9 exons on mouse chromosome 19. According to the sequence tag provided by BayGenomics, the insertional mutation in PST080 occurred within exon 9 and gene-trapped locus is expected to yield a truncated CD98hc whose C-terminal 102 amino acids are replaced with β geo (ΔCD98-β geo). The ES cells were injected into C57BL/6 blastocysts to create chimeric mice, which were bred with Black Swiss mice to generate heterozygous (CD98^Δ/+^) mice with mixed genetic background. The mice were weaned at 3 weeks of age, housed in a barrier facility with a 12-hour light/dark cycle. Eight-week-old CD98^Δ/+ ^female mice were mated with CD98^Δ/+ ^male mice at ages of 8-16 weeks to obtain homozygous mutant (CD98^Δ/Δ^) mice. Breeding pairs were examined daily for vaginal plugs and the time when vaginal plugs were detected was denoted as 0.5dpc (days post copulation). The whole uterus was removed from mated females on E7.5, E9.5, or E12.5, where implantation sites (plus embryos) and inter-implantation sites were collected separately from 3 mice on each day. Embryos were removed from the implantation sites by flushing the uterus with PBS.

### Genotyping by PCR

Genomic DNA was extracted from tail biopsies, yolk sacs, or embryos. For genotyping, two sets of primers were used. The first primer set, 5'-TTATCGATGAGCGTGGTGGTTATGC-3' and 5'- GCGCGTACATCGGGCAAATAATATC-3' were used to amplify the 681 bp region in β galactosidase. The second primer set, 5'- TGTAAGCCTCAACATGACAGTGAAG-3' and 5'-TCAGGCCACAAAGGGGAACTGTAAC-3' were used to amplify the 496 bp region in exon 9 of wild type CD98hc.

### Northern blot analysis

Mouse Mixed Tissue Northern Blot™ and Mouse Embryo Northern Blot™ were purchased from Zyagen (San Diego, CA). Specific probes for CD98hc and glyceraldehydes-3-phosphate dehydrogenase (GAPDH) were prepared by PCR amplification using the mouse kidney cDNA. The primers used were 5'-CGCCGTGGTTATCATCGTTC-3' and 5'-CGCTGGTGGATTCAAGTATGTTG-3' for CD98hc probe (596bp), and 5'-GAAGGTCGGTGTGAACGGATTT-3' and 5'TACTCCTTGGAGGCCATGTAGG-3' for GAPDH probe (996 bp). The PCR products were purified and labeled with α-^32^P dCTP using rediprime II random prime labeling system (Amersham Pharmacia Biotech, Piscataway, NJ). After standard pre-hybridization, hybridization, and washing procedures, bands were visualized via autoradiography.

### Western blotting

CD98^+/+ ^or CD98^Δ/+ ^adult mice tissues were harvested, washed twice in ice cold PBS, and dissolved in immunoprecipitation (IP) buffer (20 mM HEPES, pH 7.5, 5 mM KCl, 1.5 mM MgCl2, 1 mM EGTA, 10 mM glycerophosphate, 50 mM NaCl, 1% NP-40, 1 mM dithiothreitol, 0.2 mM phenylmethylsulfonyl fluoride, and protease inhibitor mixture (Roche, Penzberg, Germany)). After centrifugation at 14,000 rpm for 10 min at 4°C, the supernatant was boiled in sodium dodecyl sulfate (SDS) loading buffer, separated on an SDS-polyacrylamide gel, and immunoblotted with anti-CD98hc goat pAb (N-20), anti-β galactosidase rabbit pAb, anti-integrin β1 pAb, or anti-β actin mAb for internal control.

### X-gal staining

Tissues or implantation sites were harvested from sacrificed mice and fixed in 4% paraformaldehyde for 4 h at 4°C, immersed in 30% sucrose for 16 h at 4°C, and frozen in Optimal Cutting Temperature (OCT) compound (Sakura Finetek, Torrance, CA) for sectioning. β galactosidase activity was assessed by incubating 7-μm-thick sections with 1 mg/ml 5-bromo-4-chloro-3-indoyl-D-galactopyranoside (X-gal) (Sigma) for 16 h at 37°C. After counterstaining with Nuclear Fast Red (Biomeda, Foster City, CA), the sections were examined and photographed under a light microscope.

### Immunohistochemistry

The 7-μm-thick sections prepared as above were additionally fixed with acetone. The sections were incubated with anti-CD98hc rat mAb (clone H202-141, 5 μg/ml) or anti- CD98hc goat pAb (M-20, 10 μg/ml) for 1 h followed by Alexa 488-conjugated goat anti-rat IgG (dilution 1:500) or FITC-conjugated donkey anti-goat IgG (dilution 1:100) containing Hoechst33342 (dilution 1:1000) (Invitrogen), respectively, for 30 min. After the sections were thoroughly washed in PBS, they were mounted with Vectashield Mounting Medium (Vector Laboratories, Burlingame, CA) and examined under a confocal laser scanning microscope (Carl Zeiss, Jena, Germany).

### Immunocytochemistry

PST080 ES cells were plated onto coverslips coated with 0.1% gelatin in ES cell medium http://baygenomics.ucsf.edu/ and cultured for 24 h. Cells were fixed in 4% paraformaldehyde in PBS for 15 min and permeabilized with 0.4% Triton X-100 in PBS for 10 min. The cells were incubated with anti-CD98hc rat mAb (clone H202-141, 5 μg/ml) for 1 h followed by rhodamine-conjugated goat anti-rat IgG pAb (1:100 dilution) for 30 min. Then, the cells were treated with anti-β galactosidase mouse mAb (clone 5A3, 5 μg/ml) followed by Alexa488-conjugated goat anti-mouse IgG pAb (dilution 1:500) containing Hoechst33342 (1:1000 dilution). After washing, the cells were mounted with Vectashield Mounting Medium and examined under a confocal laser scanning microscope. β galactosidase activity was evaluated with X-gal staining.

### Flow cytometry

PST080 ES cells were trypsinized and washed in PBS containing 0.1% bovine serum albumin (BSA) and 0.1% NaN3. The cells were then fixed in 4% paraformaldehyde at room temperature for 10 min and washed twice in PBS. The precipitated cells (2 × 10^4 ^cells/tube) were incubated with anti-CD98hc rat mAb (clone H202-141, 50 μg/ml, 10 μl), anti-β galactosidase mouse mAb (clone 5A3, 50 μg/ml, 10 μl), or normal mouse IgG (50 μg/ml, 10 μl) (Vector) for 30 min at 4°C. After the cells were washed twice with PBS, they were incubated with Alexa-488 conjugated secondary pAbs (diluted 1:50, 20 μl) at 4°C for 30 min in the dark. The cells were then washed twice and resuspended in 300 μl of PBS. Cell surface labeling was analyzed by Alexa488 fluorescence detection using a FACScalibur (BD Biosciences). Flow cytometric data were obtained from the analysis of 5 × 10^3 ^cells per sample.

### Immunoprecipitation

PST080 ES cell lines were lysed in IP buffer. The lysates were centrifuged at 14,000 rpm for 10 min at 4°C and the supernatants were used for immunoprecipitation. Two hundred μg of total cell lysates were incubated with 4 μg of anti-integrin β1 mAb (clone 9EG7) or normal rat IgG and 10 μl of protein-G Sepharose (Amersham Biosciences, Piscataway, NJ) at 4°C for 4 h. Beads were washed three times in the same IP buffer. The immunoprecipitates were boiled in SDS loading buffer, separated on an SDS-polyacrylamide gel, and immunoblotted with anti-CD98hc goat pAb (N-20), anti-β galactosidase rabbit pAb, or anti-integrin β1 rabbit pAb.

### Frog oocyte reconstituted system

First-strand cDNA was synthesized from the total RNA extracted from PST080 ES cells using BD SMART RACE cDNA Amplification Kit (BD Biosciences, San Jose, CA) according to the manufacturer's recommendation. Wild type CD98 h, mutant CD98 (ΔCD98hc-β geo) and mutant CD98hc without β geo fusion (ΔCD98hc) were PCR amplified using the common forward primer 5'-CATCATaccggtATGAGCCAGGACACCGAAGTGGACA-3' (small letters represent a restriction site for AgeI). Reverse primers used were 5'-ATGATG**ACTAGT**TCAGGCCACAAAGGGGAACTGTAACAGC-3' for CD98hc, 5'-ATGATG**ACTAGT **TCAGAAGAACTCGTCAAGAAGGCG-3' for ΔCD98hc-β geo, and 5'-ATGATG**ACTAGT**TCAACCCCGAAGGTCACTCAGCCGCCG-3' for ΔCD98 (bold letters represent restriction sites for SpeI). PCR products were purified and cloned into T7Ts expression vector (a gift from Dr. G. J. C. Veenstra University of Nijimegen), which is based on the pGEM-4Z vector (Promega) and contains the 5'-and 3'-untranslated region of Xenopus laevis β globin gene flanking the multiple cloning sites, using AgeI and SpeI.

Flag-tagged integrin β1 was also PCR amplified and cloned into T7Ts vector. The primer used were 5'-CATCATaccggt**ATGGACTACAAAGACGATGACGATAAA**ATGAATTTGCAACTGGTT TCCTGGATTGGA-3' (small letters represent a restriction site for AgeI and underlined letters represent FLAG sequence) and ATGATG**ACTAGT**TCATTTTCCCTCATACTTCGGATTGACCAC (bold letters represent a restriction site for SpeI).

The mRNAs for CD98hc, ΔCD98hc, ΔCD98hc-β geo and FLAG-integrin β1 were synthesized with a T7 *in vitro *transcription kit (mMESSAGE mMACHINE; Ambion, Austin, TX). The mRNA for CD98hc (11.5 ng/oocyte), ΔCD98hc (11.5 ng/oocytes) or ΔCD98hc-β geo (46 ng/oocyte) were injected into the cytoplasm of 20 *Xenopus laevis *stage VI oocytes with or without the mRNA for FLAG-integrin β1 (23 ng/oocyte) [16]. The oocytes were incubated at 18°C for two days.

Some of the oocytes were fixed in 4% paraformaldehyde for 4 h at 4°C, immersed in 30% sucrose for 16 h at 4°C, and frozen in OCT compound for sectioning. 10-μm-thick sections were immunostained with anti-CD98hc rat mAb (clone H202-141, 5 μg/ml), anti-β galactosidase mouse mAb (clone 5A3, 5 μg/ml) or anti-integrin β1 rat mAb (clone 9EG7, 10 μg/ml), followed by appropriate secondary antibodies. To visualize β galactosidase activity, some of the sections were stained with X-gal as mentioned above.

Twenty oocytes from each group were lysed in IP buffer. After centrifugation at 14,000 rpm for 10 min at 4°C, a part of the supernatant was kept as input sample and the rest was used for immunoprecipitation with Ezview Red ANTI-FLAG M2 Affinity Gel (Sigma). Each lysate was incubated with 10 μl of the gel for 4 h and washed three times in the same IP buffer. The immunoprecipitates were boiled in SDS loading buffer, separated on an SDS-polyacrylamide gel, and immunoblotted with anti-FLAG M2 mouse mAb, anti-CD98hc goat pAb (N-20) or anti-β galactosidase rabbit pAb.

## Competing interests

The authors declare that they have no competing interests.

## Authors' contributions

YS and RAH designed and carried out experiments, interpreted the findings and prepared the manuscript; CL, generated CD98hc^Δ/Δ ^mice; CD and Y-BS supervised the study and prepared the manuscript. All authors read and approved the final manuscript.

## Supplementary Material

Additional file 1 **Supplement figures**. Supplement 1: Tissue distribution of CD98hc and ΔCD98hc-β geo transcripts; Supplement 2: Tissue distribution of β galactosidase activity. Click here for file
