# Supplementary material for: Extracellular domain of CD98hc is required for early murine development
Source: Cell Biosci. 2011 Feb 25;1:7. doi: 10.1186/2045-3701-1-7 (PMC3125211; doi:10.1186/2045-3701-1-7)
Supplement: Additional file 1 — Supplement figures. Supplement 1: Tissue distribution of CD98hc and ΔCD98hc-β geo transcripts; Supplement 2: Tissue distribution of β galactosidase activity. [file 2045-3701-1-7-S1.PDF]

Supplement 1

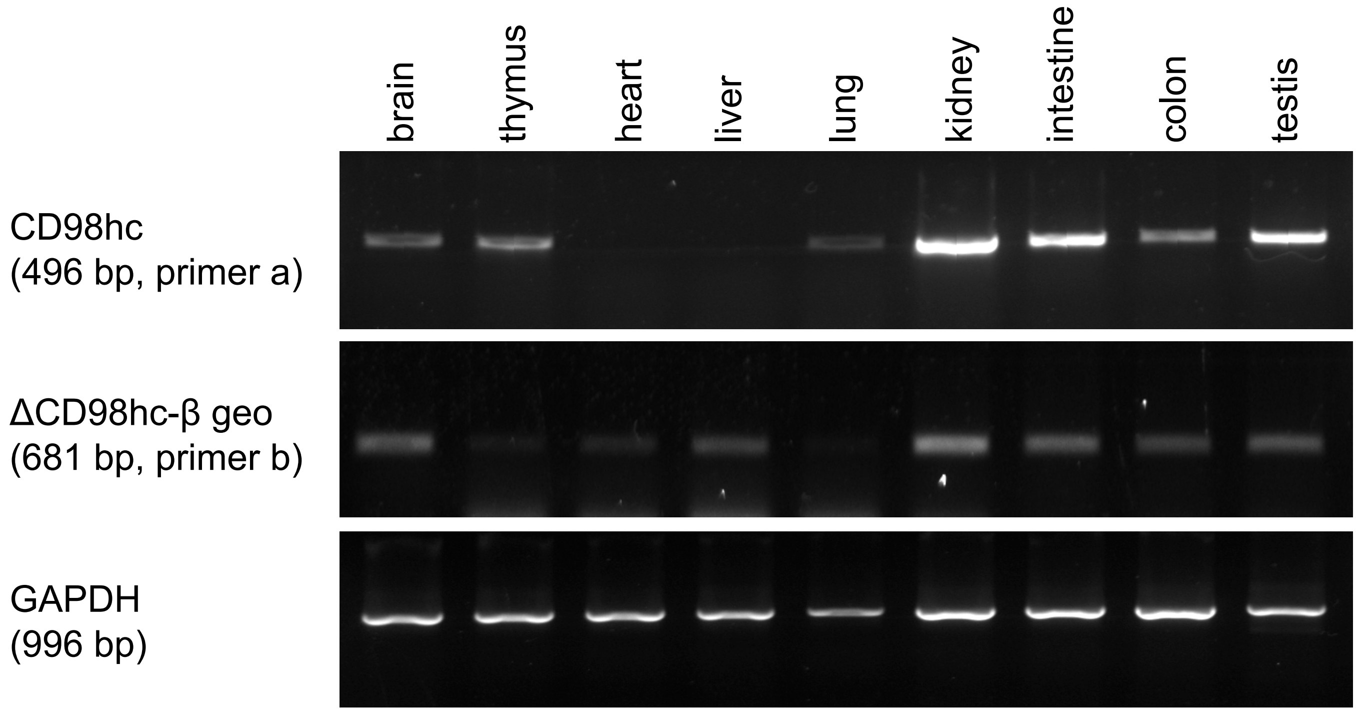

Expression of CD98hc mRNA and ΔCD98hc-β geo mRNA in various tissues obtained from a CD98hc$^{\Delta/+}$ adult mouse. Primer set A, which amplifies whole translated region of exon 9 (496 bp), yields the expected PCR product from wild type CD98hc allele (+). Primer set B, which amplifies a part of β-geo sequence (681 bp), yields PCR products only from the mutant allele (Δ). Note that primer set A yields the 496-bp band that shows the presence of wild type CD98hc allele in all the offspring. GAPDH represents control expression. GAPDH fwd primer; GAAGGTCGGTGTGAACGGATTT, GAPDH Rev primer; TACTCCTTGGAGGCCATGTAGG.

Supplement 2

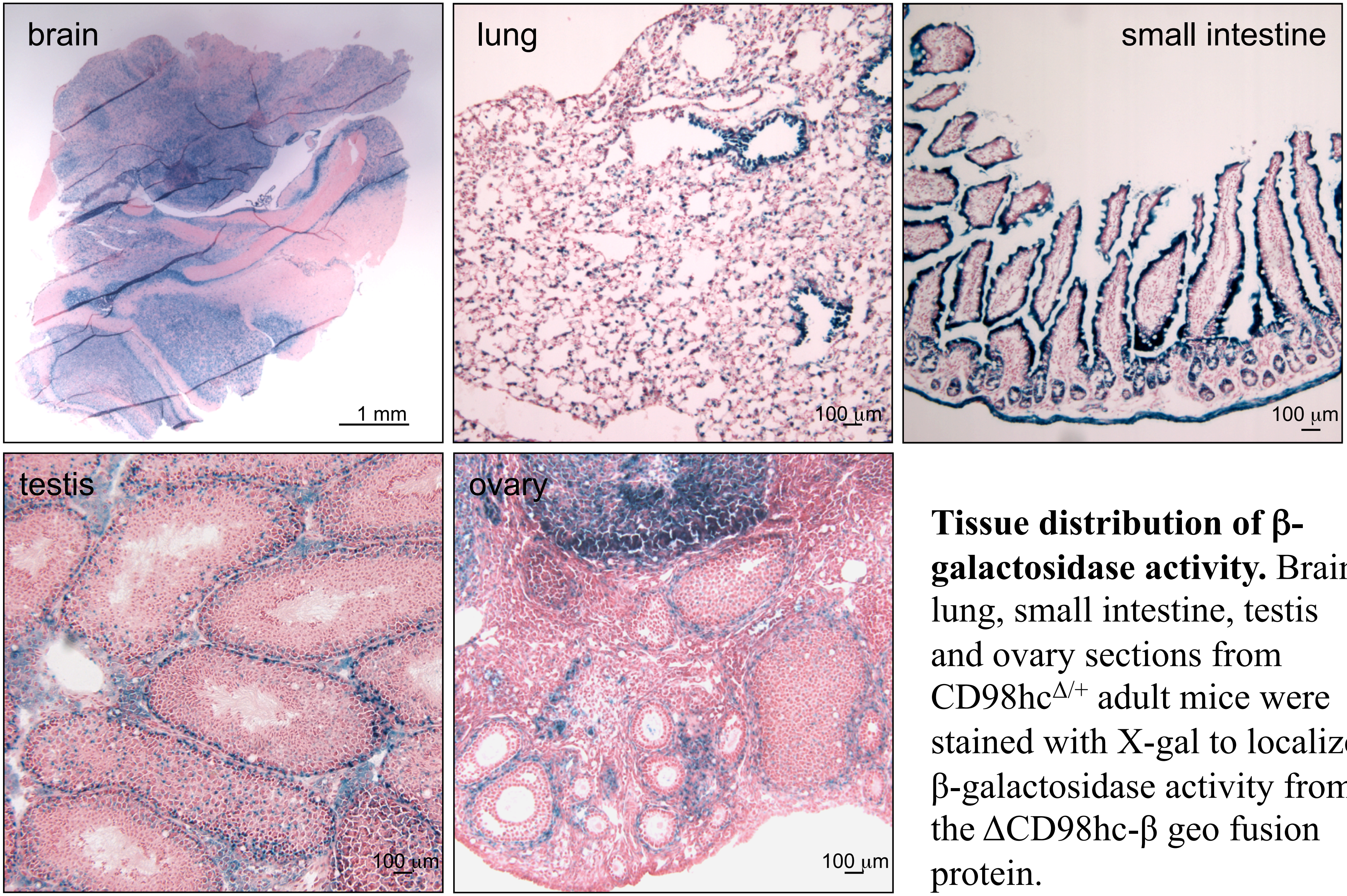

Tissue distribution of β-galactosidase activity. Brain, lung, small intestine, testis and ovary sections from CD98hc$^{\Delta/+}$ adult mice were stained with X-gal to localize β-galactosidase activity from the ΔCD98hc-β geo fusion protein.
